# Computed tomography-based quantitative scoring system for rheumatoid arthritis–associated interstitial lung disease: a retrospective diagnostic accuracy study for progressive fibrosis detection

**DOI:** 10.1007/s10067-025-07511-y

**Published:** 2025-06-09

**Authors:** Mehmet Cihan Karacaoğlu, Betül Kızıldağ, Burak Okyar, Gözde Yıldırım Çetin, Adem Doğaner, Nurhan Atilla

**Affiliations:** 1 Department of Radiology, Sütçü İmam University, Kahramanmaraş, Turkey; 2 Department of Rheumatology, Adana City Training and Research Hospital, Adana, Turkey; 3 Department of Rheumatology, Sütçü İmam University, Kahramanmaraş, Turkey; 4 Department of Biostatistics, Sütçü İmam University, Kahramanmaraş, Turkey; 5 Department of Pulmonology, Sütçü İmam University, Kahramanmaraş, Turkey; 6 Department of Radiology, Etlik City Hospital, Ankara, Turkey

**Keywords:** Computer-assisted image analysis, Interstitial lung diseases, Multidetector computed tomography, Pulmonary function tests, Rheumatoid arthritis

## Abstract

**Objectives:**

To investigate the ability of quantitative parameters to assess the severity of rheumatoid arthritis (RA)–associated interstitial lung disease (ILD).

**Methods:**

Thorax CT images and pulmonary function tests of RA patients followed up in a tertiary reference center were retrospectively examined. Radiologically, patients were divided into two groups as limited and extensive ILD, using two semiquantitative scoring systems. Two different methods (Method 1, Method 2) were used for quantitative image analysis, in which different Hounsfield unit values were selected as thresholds. Spearman’s correlation test was used to evaluate the relationship between variables. The diagnostic performance of quantitative methods for the ability to distinguish limited ILD from extensive ILD was calculated using ROC analysis.

**Results:**

Forty-four patients, 29 female and 15 male, were included in the study. A significant correlation was found between diffusion capacity of the lungs carbonmonoxide (DLCO) and total lung capacity (TLC), which are clinical markers, and both quantitative methods (*p* < 0.001). In terms of the performance of diagnostic tests, the power of Method 1 and Method 2 to distinguish limited and extensive disease classified based on semiquantitative scores was found to be strong (Method 1 AUC = 0.945, Method 2 AUC = 0.785 based on “ILD score”; Method 1 AUC = 0.911, Method 2 AUC = 0.700 based on “modified coarseness of reticular disease (MCRD) score”).

**Conclusion:**

This study demonstrates that quantitative methods (Method 1 [− 700 to − 200 HU] and Method 2 [− 800 to − 500 HU]) play a significant role in assessing the severity of RA-associated ILD (RA-ILD). Both methods showed significant correlations with pulmonary function tests (DLCO [*r*_1_ = − 0.338, *p*_1_ = 0.025; *r*_2_ = − 0.452, *p*_2_ = 0.002] and TLC [*r*_1_ = − 0.567, *p*_1_ < 0.001; *r*_2_ = − 0.576, *p*_2_ < 0.001), with Method 1 (AUC_ILD_ = 0.945 and AUC_MCRD_ = 0.911) demonstrating superior performance in distinguishing between limited and extensive ILD. Our findings suggest that Hounsfield unit threshold–based quantitative CT analysis may serve as a more objective and reproducible alternative to semiquantitative systems (e.g., Goh score) in the standard evaluation of RA-ILD. Specifically, Method 1 may enable early detection of progression by mechanism, e.g., “tracking subtle density changes.” However, further validation in multicenter prospective cohorts is needed to address limitations such as bias.

**Table:** 

**Key Points** • *Interobserver and intraobserver variability poses a significant challenge in the objective assessment of rheumatoid arthritis–associated interstitial lung disease*. • *User independent quantitative methods can be used instead of user-dependent semiquantitative methods in assessing rheumatoid arthritis–associated interstitial lung disease*. • *Quantitative computed tomography analysis enables precise stratification of disease severity in rheumatoid arthritis-associated interstitial lung disease, distinguishing limited interstitial lung disease from extensive interstitial lung disease, which has a poor prognosis*.

## Introduction

The global prevalence rate of rheumatoid arthritis (RA) was reported as 0.21% in 2020 [1]. RA-associated interstitial lung disease (RA-ILD) is a source of substantial morbidity and mortality for affected patients [2]. The progression or regression of ILD findings is of prognostic significance and determines whether treatment should be continued or modified [3, 4]. RA-ILD is often asymptomatic in early stages, leading to delayed diagnosis until irreversible fibrosis develops. Current gold-standard diagnostic methods—such as high-resolution CT (HRCT) and pulmonary function tests (PFTs)—are limited by interobserver variability in semiquantitative methods (visual scoring) and the inability of PFTs to detect early parenchymal changes. This underscores the need for objective, reproducible tools to quantify disease severity and monitor progression [5, 6]. Evaluation of interstitial involvement, which significantly impacts prognosis, requires consistent results across different evaluators. An ideal assessment method should also be rapid and easy to implement. Quantitative methods are especially appropriate for ILD evaluation since they remove interobserver variability and decrease reliance on the evaluator’s experience.

Until now, several different quantitative lung assessment (QLA) methods have been developed using various histogram-based ILD methods and threshold values for detecting areas of pathological ground-glass or fibrosis in ILD. No consensus exists on optimal histogram thresholds for RA-ILD severity stratification, hindering clinical adoption. By establishing threshold-based criteria for disease severity, we aim to bridge the gap between research tools and routine clinical practice [7–25].

In this study, we adapted two quantitative methods previously validated for systemic sclerosis-associated ILD. These methods have demonstrated strong correlation with visual assessment scores in previous studies. Although quantitative CT (QCT) methods have been proposed for ILD assessment in systemic sclerosis, their applicability to RA-ILD remains underexplored. Given the histopathological similarities between RA-ILD and systemic sclerosis-associated ILD [26], we hypothesized that QCT methods validated in the latter could be transposed to RA-ILD with comparable accuracy [9, 10].There are a few studies in the literature based on a quantitative analysis of the ILD involvement associated with RA. There are studies reported on the correlation between quantitative CT evaluation and visual CT evaluation using a study population of RA patients. In one study, − 800 Hounsfield unit (HU) histogram threshold was defined [13], while the other study was conducted using pattern or texture recognition method [7]. Unlike prior RA studies, we evaluated two distinct histogram threshold–based approaches—Method 1 and Method 2—against both visual scoring and functional parameters (PFTs, DAS28-CRP) to establish a clinically relevant correlation.

This study aims to validate two histogram-based QCT methods for RA-ILD severity assessment against semiquantitative visual scores and PFTs, and identify optimal HU thresholds to distinguish limited vs. extensive disease, thereby providing a standardized tool for early progression detection.

## Materials and methods

### Study population

This study is a cross-sectional and retrospective original research article. This study conducted after obtaining ethical committee approval with decision number 03 on 01.02.2022. From January 2016 to December 2021, centrally followed patients with known RA and associated ILD having HRCT and PFTs were included in the study. Among the patients admitted to the rheumatology outpatient clinic, the 2010 ACR/EULAR classification criteria were used to diagnose RA.

Demographic data of patients (age, sex), duration of illness, history of smoking, PFTs, autoantibody profile of RA patients, DAS28-CRP disease severity score, and medications used were recorded.

Among patients over the age of 18 with ILD associated with RA, HRCT analysis was performed. Patients who underwent HRCT and had PFTs within the past 2 weeks were included in the study. Patients with moderate to severe pulmonary arterial hypertension with an average pulmonary artery pressure of 30 mmHg or higher, pulmonary edema, active pulmonary infection or lung mass presence, history of thoracic surgery, severe tuberculosis, prior thoracic radiotherapy, and major motion artifacts that could affect HRCT evaluation were excluded from the study. In addition, patients who could not perform the PFTs properly were excluded from the study.

We used power analysis to determine the sample size. In the study, it was planned to include a total of 44 patients, 22 + 22, in the calculation made by taking into account the means of 87 and 78 and the standard deviations of 8 and 12 for the FEV_1_ parameter in the reference study [27] with a test power of 0.80 at the alpha 0.05 first type error and beta 0.20 s type error level.

### Pulmonary function tests

Forced vital capacity (FVC), forced expiratory volume in 1 s (FEV_1_), FEV_1_/FVC, total lung capacity (TLC), and diffusion capacity of the lungs for carbon monoxide (DLCO) were measured. PFTs were obtained using widely accepted techniques and the results were expressed as percentages (%) [28]. PFTs were performed in accordance with the American Thoracic Society (ATS) criteria/guidelines [29].

### HRCT reviews

HRCT images are performed using a multi-detector (320) CT system (Aquilion ONE ViSION edition; Canon Medical Systems Corporation) obtained by helical scanning from the apex of the lung to its bases. Assessments were performed on CT devices that had undergone calibration or interscan variability checks.

The “acquisition” parameters were as follows: detector width 80 × 0.5 mm, tube voltage (120 kV), tube current 250–300 mAs, slice thickness 3 mm, slice interval 1.5 mm, rotation time 0.35, pitch factor 1.388, field of view variable 35–45 cm (example 40*40 cm).

We used a phantom with a diameter of 32 cm to represent an adult’s body, and the mean CTDIvol and DLP values were 4.8 mGy and 182.7 mGycm, respectively.

### Semiquantitative image analysis

Two people scanned at all the HRCT images, without knowing about the clinical findings, PFTs results, or quantitative measurements. To minimize bias, HRCT evaluations were conducted in a blinded manner, and interobserver agreement was analyzed. The lung window settings had a window center of − 500 to − 600 HU and a window width of 1600 HU.

HRCT images were evaluated by two radiologists, a thoracic radiologist (B.K.) with 20 years of experience and a radiology assistant (M.C.K) with 4 years of experience, according to the ILD prevalence score defined by Goh et al. [30] and according to the modified coarseness of reticular disease (MCRD) scoring system [31]. CT images were scored at five different cross-sectional levels: the origin of the large vessels, the main carina, the pulmonary venous junction, the middle of the third and fifth sections, and just above the right hemidiaphragm.

**Fig. 1:**
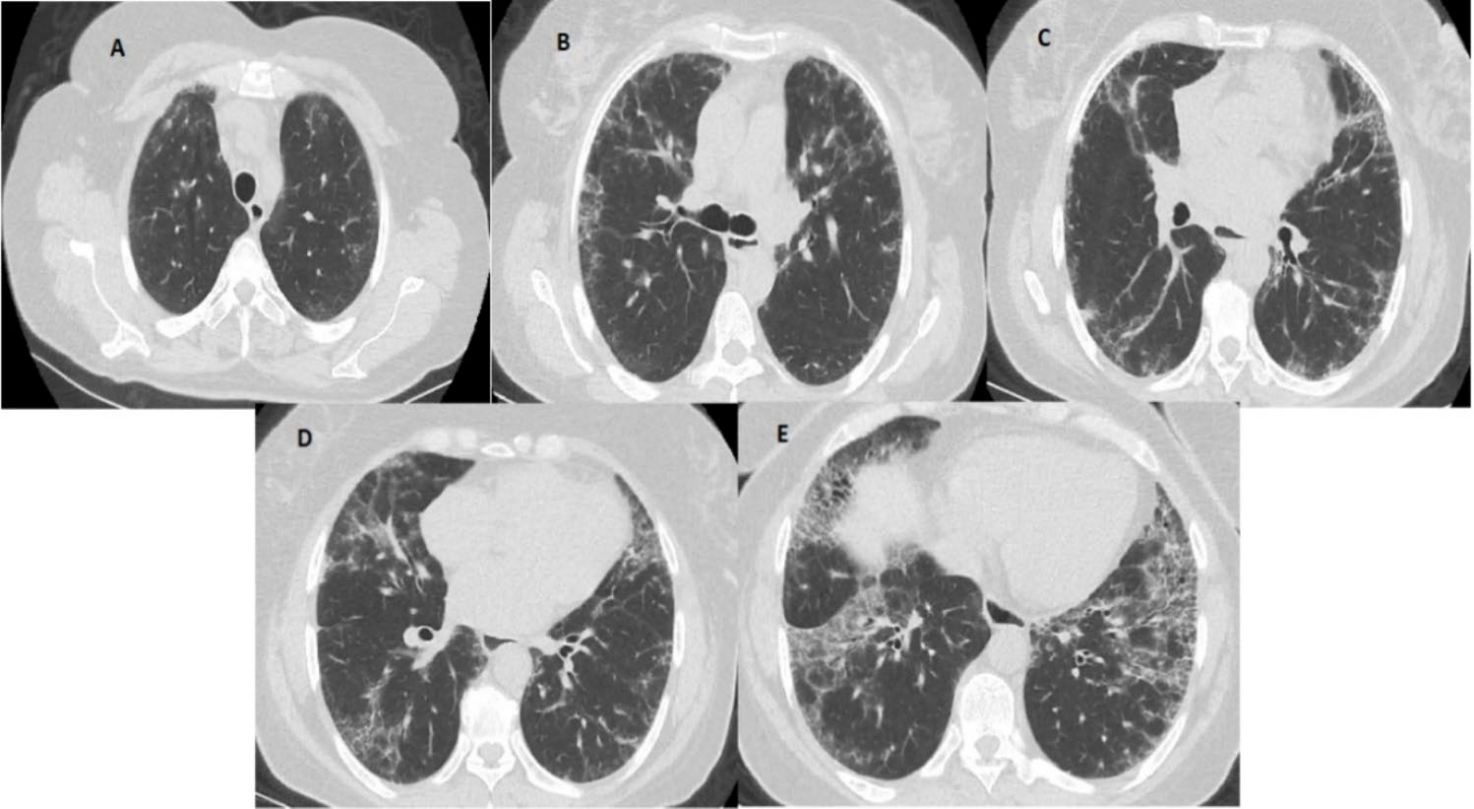
An example of a semiquantitative HRCT assessment. Five levels of HRCT images. **A** Origin of great vessels (ILD score 5% and MCRD score 2). **B** Main carina (ILD score 15% and MCRD score 2). **C** Pulmonary venous confluence (ILD score of 30% and MCRD score of 2). **D** Halfway between the third and fifth sections (ILD score 45% and MCRD score 2). **E** Immediately above the right hemidiaphragm (ILD score of 65% and MCRD score of 3)

According to the “ILD score,” the prevalence of the disease was estimated as a percentage of the total area, with 5% and multiples in each of the five CT sections. One-fifth of the total score on five CT sections was considered the total lung involvement rate according to the ILD prevalence score. Two groups were formed as patients with limited (< 20%) and extensive (≥ 20%) ILD. According to the “MCRD score,” the prevalence of the disease was estimated as follows: 0, normal; 1, ground-glass opacity alone; 2, fine intralobular fibrosis; 3, microcystic honeycomb appearance (≤ 4 mm); 4, macrocystic honeycomb appearance (> 4 mm). The highest score in each section was taken into account, MCRD was calculated to be the total score for all five levels, MCRD ≤ 10 was considered mild reticulation; and MCRD > 10 was considered severe reticulation (Fig. 1).

### Quantitative image analysis

Blind to PFTs findings, clinical data, and semiquantitative measurement results, a radiology resident (M.C.K.) with 4 years of experience was present at work (Vitrea; Canon Medical Systems Corporation), which was performed fully automatically in software called “Lung Density Analysis.” We calculated the descriptive parameters of the quantitative analysis at various CT attenuation values [8–13, 15, 16, 18].

A trained radiologist performed all histogram-based quantitative assessments in different sessions. We did not investigate the agreement between observers due to the nature of quantitative measurement. When necessary, we performed minimal user intervention to exclude pulmonary vessels, the esophagus, trachea, and main bronchi.

We used two different quantitative methods, previously used in the literature to evaluate ILD in systemic sclerosis patients and reported as successful, to calculate the level of ILD in RA patients.

We used − 950 HU as the lowest density limit value of the lung parenchyma to exclude air cyst volume and emphysema in the lung.

### Histogram-based methods

In the “Lung Density” program, low density volume index (LD Index (%)), upper lung low density volume index (Up. Lung LDI (%)), lower lung low density volume index (Lo. Lung LDI (%)), Up/Low Ratio, and 15th percentile density (PD15) are calculated based on the threshold values automatically assigned to low (− 1024 to − 920 HU voxels), medium (− 920 to − 720 HU voxels), and high (− 720 to 0 HU voxels) density values. We calculate the LD index (%) as [low density volume/(low density volume + medium density volume)]. PD15 is the 15 th percentile density (g/l).

**Fig. 2:**
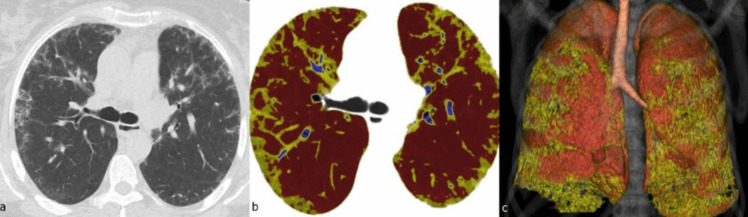
A case of quantitative imaging (patient with HRCT images shown in Fig. 1). In the case study, involvement in the form of peripherally located patchy thin reticulation is observed in the axial HRCT section passing through the carina level (**a**). In the quantitative analysis of the cross-section passing through the same level, the threshold values between − 700 and − 200 HU, which correspond to the regions held, are represented in yellow (**b**). Three-dimensional and color quantitative analysis of the same patient is monitored (**c**)

Method 1: We selected all voxels between − 950 and − 200 HU to calculate lung volume. All voxels between − 700 and − 200 HU were calculated as ILD, as defined by Salaffi et al. [10]. Method 2: All voxels between − 950 and − 250 HU were selected to calculate lung volume. All voxels between − 800 and − 500 HU were calculated as ILD as defined by Yabuuchi et al. [9]. The quantitative assessment score was calculated as (quantitative ILD volume/quantitative total lung volume) × 100 (Fig. 2).

### Statistical analysis

The Shapiro–Wilk test examined the conformity of the variables to the normal distribution in the data evaluation. The comparison of two groups in variables that conform to the normal distribution was evaluated with an independent sample *t* test, and the comparison of two groups in variables that do not conform to the normal distribution was evaluated with a Mann–Whitney *U* test. Comparisons between three or more groups in variables that did not conform to the normal distribution were made with the Kruskal–Wallis *H* test. Frequency distribution differences between qualitative variables were evaluated by the chi-square test and the exact test. We evaluated the relationship between quantitative variables that did not conform to the normal distribution using the Spearman correlation test. Statistical parameters were expressed as mean ± standard deviation, median (25% quartile–75% quartile), ratio (%), and frequency (*n*). Statistical significance was accepted as *p* < 0.05. We evaluated the data using IBM SPSS version 22 (IBM SPSS for Windows version 22, IBM Corparation, Armonk, New York, United States) and R.3.3.2 software.

## Results

**Fig. 3:**
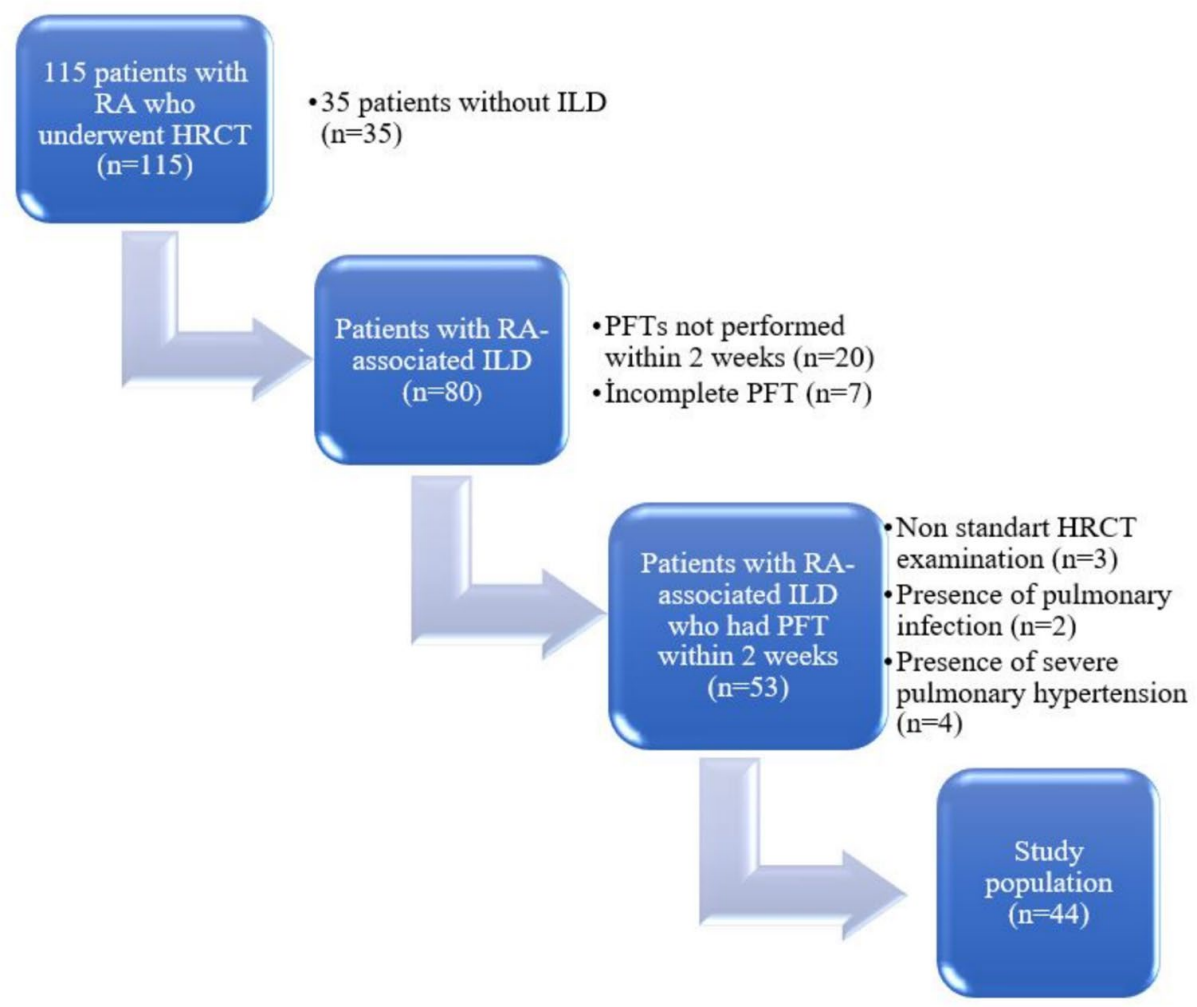
Flowchart of the study population

HRCT was performed in 115 patients with RA, and ILD was detected in 80 cases. Of the 53 patients who underwent PFTs within 2 weeks, they were evaluated. A total of 44 patients were included in the study after excluding those with severe motion artifacts (*n* = 3), pulmonary infection (*n* = 2), and severe pulmonary hypertension (*n* = 4) (Fig. 3).

Forty-four patients, 29 female and 15 male, were included in the study. The baseline characteristics of the patients are shown in Table 1. There was a distributional difference between smokers and non-smokers according to gender (*p* = 0.034) (Table 1).

**Table 1:** Baseline characteristics of the patients with RA

		Males	Females	*p*
Age, median (Q1–Q3)		53.00 (51.00–71.00)	65.00 (58.00–69.00)	0.25
Smoking status	Not smoke, *n* (%)	12.00 (80.00)	29.00 (100.00)	0.034*
	Smokes, *n* (%)	3.00 (20.00)	0.00 (0.00)	
Disease duration, median (Q1–Q3)		9.00 (1.00–15.00)	10.00 (8.00–17.00)	0.17
Pattern	DIP, *n* (%)	1.00 (6.67)	0.00 (0.00)	0.12
	LIP, *n* (%)	0.00 (0.00)	2.00 (6.90)	
	NSIP, *n* (%)	3.00 (20.00)	13.00 (44.83)	
	UIP, *n* (%)	11.00 (73.33)	14.00 (48.28)	
ACPA, median (Q1–Q3)		173.00 (3.00–300.00)	92.00 (5.10–290.00)	0.85
RF, median(Q1–Q3)		140.00 (13.20–683.00)	128.00 (32.20–262.00)	0.61
DAS28-CRP, median(Q1–Q3)		2.79 (1.70–4.31)	2.16 (1.60–2.84)	0.33
SQLA(ILD), median(Q1–Q3)		10.00 (3.00–18.00)	3.00 (0.00–22.00)	0.59
SQLA(MCRD), median(Q1–Q3)		10.00 (6.00–16.00)	4.00 (0.00–12.00)	0.70
FVC% (PFT), mean ± SD		78.40 (14.36)	74.45 (26.43)	0.59
FEV_1_% (PFT), mean ± SD		79.00 (12.81)	70.55 (23.43)	0.20
TLC, mean ± SD		81.53 (15.10)	84.93 (15.16)	0.48
DLCO, median (Q1–Q3)		79.00 (45.00–92.00)	73.00 (57.00–89.00)	0.92

Median (Q1–Q3): the median and interquartile range.

Mean ± SD: the mean and standard deviation.

*p*-value: The *p*-value indicating the statistical significance.

*n* (%): Number and percentage of patients.

*indicates statistical significance (*p* < 0.05).

*DIP*, desquamative interstitial pneumonia; *LIP*, lymphoid interstitial pneumonia; *NSIP*, nonspecific interstitial pneumonia; *UIP*, usual interstitial pneumonia; *ACPA*, anti-citrullinated protein antibody; *RF*, rheumatoid factor; *DAS*, disease activity score; *CRP*, C reactive protein; *SQLA*, semiquantitative lung assessment; *ILD*, interstitial lung disease; *MCRD*, modified coarseness of reticular disease; *PFT*, pulmonary function test; *FVC*, forced vital capacity; *FEV*_*1*_, forced expiratory volume in 1 s; *TLC*, total lung capacity; *DLCO*, diffusion capacity of the lungs for carbon monoxide.

In the semiquantitative evaluation obtained with the ILD and MCRD scores, comparing limited and extensive disease, a significant difference was found in terms of the mean quantitative measurements of Method 1 (*p* < 0.001 for the ILD score and *p* = 0.001 for the MCRD score) and Method 2 (*p* = 0.005 for the ILD score and *p* = 0.038 for the MCRD score) (Table 2, Table 3).

**Table 2:** Characteristics of RA patients with limited or extensive ILD according to ILD score

Characteristic	Limited ILD	Extensive ILD	*p*-value
Age, median (Q1–Q3)	62.0 (52.0–69.0)	65.0 (52.0–69.0)	0.88
Smoking status			0.56
Not smoke, *n* (%)	30 (90.90)	11 (100.00)	
Smokes, *n* (%)	3 (9.10)	0 (0.00)	
Disease duration, median (Q1–Q3)	9.0 (4.0–16.0)	12.0 (8.0–17.0)	0.36
Pattern			0.70
- DIP, *n* (%)	1 (3.00)	0 (0.00)	
- LIP, *n* (%)	1 (3.00)	1 (9.10)	
- NSIP, *n* (%)	13 (39.40)	3 (27.30)	
- UIP, *n* (%)	18 (54.50)	7 (63.60)	
ACPA, median (Q1–Q3)	162.00 (16.00–290.00)	12.00 (3.00–300.00)	0.35
RF, median (Q1–Q3)	162.00 (38.90–291.00)	80.60 (12.00–604.00)	0.27
DAS28-CRP, median (Q1–Q3)	2.70 (1.80–4.16)	1.60 (1.40–2.10)	0.003*
SQLA (MCRD ≤ 10), median (Q1–Q3)	4.0 (1.0–8.0)	15.0 (13.0–19.0)	*p* < 0.001*
SQLA median (Q1–Q3)	2.0 (1.0–11.0)	29.0 (23.0–56.0)	*p* < 0.001*
FVC% (PFT), mean ± SD	76.30 ± 23.90	74.20 ± 20.70	0.80
FEV_1_% (PFT), mean ± SD	74.10 ± 22.30	71.50 ± 15.30	0.72
FEV_1_/FVC% (PFT), mean ± SD	103.60 ± 11.70	105.90 ± 12.40	0.58
TLC, mean ± SD	86.10 ± 14.60	76.90 ± 14.90	0.08
DLCO, median (Q1–Q3)	79.0 (60.0–92.0)	71.0 (30.0–81.0)	0.07
LD Index (%), median (Q1–Q3)	0.06 (0.02–0.13)	0.02 (0.01–0.08)	0.12
PD15 (g/l), median (Q1–Q3)	98.0 (83.0–119.0)	117.0 (98.0–156.0)	0.07
Up. Lung LDI (%), median (Q1–Q3)	0.07 (0.01–0.14)	0.01 (0.01–0.09)	0.17
Lo. Lung LDI (%), median (Q1–Q3)	0.07 (0.02–0.14)	0.04 (0.01–0.06)	0.11
Up/Low Ratio, median (Q1–Q3)	0.92 (0.61–1.40)	0.86 (0.55–1.34)	0.70
Method 1, median (Q1–Q3)	12.13 (8.66–14.74)	25.37 (18.93–37.73)	*p* < 0.001*
Method 2, median (Q1–Q3)	25.21 (18.05–33.06)	36.26 (33.27–62.91)	0.005*

Median (Q1–Q3): the median and interquartile range.

Mean ± SD: the mean and standard deviation.

p-value: the p-value indicating the statistical significance.

n (%): number and percentage of patients.

*indicates statistical significance (*p* < 0.05).

*DIP*, desquamative interstitial pneumonia; *LIP*, lymphoid interstitial pneumonia; *NSIP*, nonspecific interstitial pneumonia; *UIP*, usual interstitial pneumonia; *ACPA*, anti-citrullinated protein antibody; *RF*, rheumatoid factor; *DAS*, disease activity score; *CRP*, C reactive protein; *SQLA*, semiquantitative lung assessment; *ILD*, interstitial lung disease; *MCRD*, modified coarseness of reticular disease; *PFT*, pulmonary function test; *FVC*, forced vital capacity; *FEV*_*1*_, forced expiratory volume in 1 s; *TLC*, total lung capacity; *DLCO*, diffusion capacity of the lungs for carbon monoxide; *LD Index*, low density volume index; *PD15*, 15th percentile density; *Up. Lung LDI*, upper lung low density volume index; *Lo. Lung LDI*, lower lung low density volume index.

**Table 3:** Characteristics of RA patients with limited or extensive ILD according to MCRD score

Characteristic	Limited ILD	Extensive ILD	*p*-value
Age, median (Q1–Q3)	62.0 (52.0–69.0)	65.0 (52.0–69.0)	0.96
Smoking status			0.54
- Does not smoke, *n* (%)	28 (90.30)	13 (100.00)	
- Smokes, *n* (%)	3 (9.70)	0 (0.00)	
Disease duration, median (Q1–Q3)	10.0 (4.0–17.0)	9.0 (8.0–12.0)	0.98
Pattern			0.33
- DIP, *n* (%)	1 (3.20)	0 (0.00)	
- LIP, *n* (%)	2 (6.50)	0 (0.00)	
- NSIP, *n* (%)	13 (41.90)	3 (23.10)	
- UIP, *n* (%)	15 (48.40)	10 (76.90)	
ACPA, median (Q1–Q3)	162.00 (16.00–300.00)	12.00 (3.00–180.00)	0.26
RF, median (Q1–Q3)	162.00 (42.20–430.00)	30.70 (12.00–280.00)	0.11
DAS28-CRP, median (Q1–Q3)	2.70 (1.80–4.16)	1.60 (1.40–2.10)	0.012*
SQLA (MCRD ≤ 10), median (Q1–Q3)	1.0 (1.0–8.0)	16.0 (15.0–19.0)	*p* < 0.001*
SQLA, median (Q1–Q3)	1.0 (1.0–10.0)	23.0 (22.0–46.0)	*p* < 0.001*
FVC% (PFT), mean ± SD	77.10 ± 24.50	72.60 ± 19.30	0.56
FEV_1_% (PFT), mean ± SD	74.20 ± 23.10	71.50 ± 13.90	0.70
FEV_1_/FVC% (PFT), mean ± SD	102.90 ± 11.80	107.30 ± 11.60	0.26
TLC, mean ± SD	86.40 ± 14.50	77.50 ± 14.90	0.07
DLCO, median (Q1–Q3)	75.0 (57.0–92.0)	73.0 (38.0–92.0)	0.55
LD Index (%), median (Q1–Q3)	0.06 (0.02–0.13)	0.06 (0.01–0.11)	0.55
PD15 (g/l), median (Q1–Q3)	102.0 (83.0–120.0)	112.0 (96.0–127.0)	0.46
Up. Lung LDI (%), median (Q1–Q3)	0.06 (0.01–0.14)	0.05 (0.01–0.11)	0.57
Lo. Lung LDI (%), median (Q1–Q3)	0.06 (0.02–0.10)	0.05 (0.01–0.12)	0.54
Up/Low Ratio, median (Q1–Q3)	0.99 (0.61–1.40)	0.86 (0.55–1.34)	0.69
Method 1, median (Q1–Q3)	12.13 (8.33–14.74)	23.57 (18.93–31.99)	0.001*
Method 2, median (Q1–Q3)	26.26 (17.15–36.79)	33.42 (27.74–48.91)	0.038*

Median (Q1–Q3): the median and interquartile range.

Mean ± SD: the mean and standard deviation.

*p*-value: The *p*-value indicating the statistical significance.

*n* (%): number and percentage of patients.

*indicates statistical significance (*p* < 0.05).

*DIP*, desquamative interstitial pneumonia; *LIP*, lymphoid interstitial pneumonia; *NSIP*, nonspecific interstitial pneumonia; *UIP*, usual interstitial pneumonia; *ACPA*, anti-citrullinated protein antibody; *RF*, rheumatoid factor; *DAS*, disease activity score; *CRP*, C reactive protein; *SQLA*, semiquantitative lung assessment; *ILD*, interstitial lung disease; *MCRD*, modified coarseness of reticular disease; *PFT*, pulmonary function test; *FVC*, forced vital capacity; *FEV*_*1*_, forced expiratory volume in 1 s; *TLC*, total lung capacity;

*DLCO*, diffusion capacity of the lungs for carbon monoxide; *LD Index*, low density volume index; *PD15*, 15th percentile density; *Up. Lung LDI*, upper lung low density volume index; *Lo. Lung LDI*, lower lung low density volume index.

The evaluation of agreement between semiquantitative methods revealed a Kappa coefficient of 0.771 and a *p*-value of 0.01, indicating significant agreement between the methods in determining limited and extensive disease.

In our study, semiquantitative evaluation was found to show strong agreement according to the ILD score (*κ* = 0.840) and near-perfect agreement according to the MCRD score (*κ* = 0.892) in the consensus evaluation of two radiologists.

A negative correlation was found between “Method 1 and Method 2” and DLCO. A positive correlation was found between Method 1 and semiquantitative methods. No correlation was found between quantitative methods and PFTs (FEV_1_, FVC) (Table 4).

**Table 4:** Correlation between quantitative results and semiquantitative results-PFTs

		SQLA (MCRD)	SQLA (ILD)	FVC% (PFT)	FEV_1_% (PFT)	TLC	DLCO
LD Index (%)	*r*	0.081	0.005	0.054	0.128	0.263	0.239
	*p*	0.600	0.976	0.727	0.407	0.084	0.118
PD15 (g/l)	*r*	− 0.067	0.041	− 0.104	− 0.201	− 0.395	− 0.359
	*p*	0.664	0.794	0.501	0.191	0.008*	0.017*
Up. Lung LDI(%)	*r*	0.079	0.020	0.070	0.130	0.223	0.230
	*p*	0.610	0.898	0.653	0.401	0.145	0.133
Lo. Lung LDI(%)	*r*	0.067	− 0.030	− 0.025	0.057	0.258	0.226
	*p*	0.667	0.844	0.874	0.716	0.090	0.140
Up/Low Ratio	*r*	0.092	0.126	0.060	0.093	0.003	0.037
	*p*	0.554	0.413	0.696	0.548	0.983	0.811
Method 1	*r*	0.556	0.612	− 0.145	− 0.125	− 0.567	− 0.338
	*p*	*p* < 0.001*	*p* < 0.001*	0.346	0.418	*p* < 0.001*	0.025*
Method 2	*r*	0.160	0.252	− 0.148	− 0.208	− 0.576	− 0.452
	*p*	0.299	0.099	0.338	0.175	*p* < 0.001*	0.002*

*indicates statistical significance (*p* < 0.05)

*SQLA*, semiquantitative lung assessment; *MCRD*, modified coarseness of reticular disease; *ILD*, interstitial lung disease; *FVC*, forced vital capacity; *FEV*_*1*_, forced expiratory volume in 1 s; *TLC*, total lung capacity; *DLCO*, diffusion capacity of the lungs for carbon monoxide; *LD Index*, low density volume index; *Up. Lung LDI*, upper lung low density volume index; *Lo. Lung LDI*, lower lung low density volume index; *PD15*, 15th percentile density

**Fig. 4:**
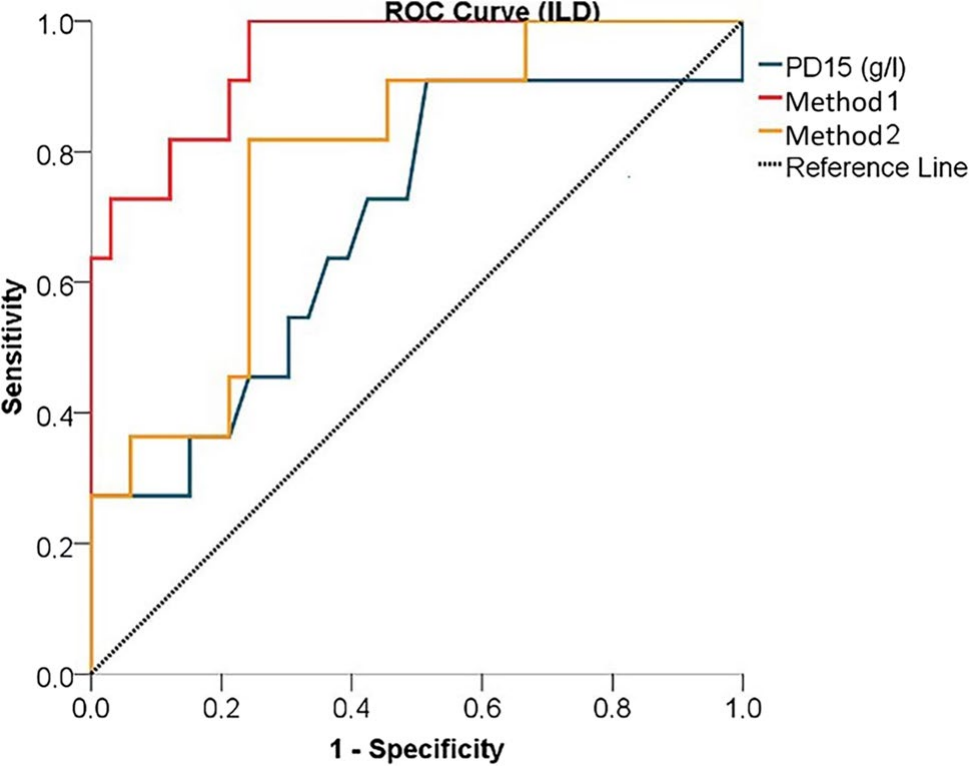
The power of quantitative methods to distinguish limited and extensive disease according to the ILD score

In terms of the performance of diagnostic tests, the power to distinguish between limited and extensive disease according to the ILD score, PD15 was found to be weak, while Method 1 and Method 2 were found to be strong. The strongest test was found to be Method 1 (Method 1 AUC = 0.945 *p* < 0.001, Method 2 AUC = 0.785 *p* = 0.005) (Fig. 4, Table 5). When the ROC curves are plotted according to the ILD score, the cutoff value in distinguishing between limited and extensive disease, 16.85 was found for Method 1 and 33.16 for Method 2 (Table 5).

**Table 5:** The ability of quantitative methods to distinguish between limited and extensive disease based on ILD and MCRD scores

	AUC	STD error	%95 CI lower	%95 CI upper	*p*	S1	S2	Cutoff
LD Index (%) ILD LD Index (%) MCRD	0.658 0.558	0.102 0.105	0.458 0.353	0.859 0.763	0.12 0.55			
Up. Lung LDI (%) ILD Up. Lung LDI (%) MCRD	0.640 0.555	0.108 0.108	0.429 0.344	0.852 0.766	0.17 0.57			
Lo. Lung LDI (%) ILD Lo. Lung LDI (%) MCRD	0.664 0.560	0.097 0.100	0.474 0.364	0.854 0.755	0.11 0.54			
Up/Low Ratio ILD Up/Low Ratio MCRD	0.540 0.538	0.101 0.099	0.343 0.344	0.737 0.733	0.69 0.69			
PD15 (g/l) ILD PD15 (g/l) MCRD	0.687 0.571	0.097 0.104	0.497 0.368	0.877 0.774	0.07 0.46			
Method 1 ILD Method 1MCRD	0.945 0.911	0.034 0.051	0.879 0.810	1.000 1.000	< 0.001* < 0.001*	0.909 0.923	0.788 0.774	16.85 14.99
Method 2 ILD Method 2 MCRD	0.785 0.700	0.075 0.084	0.637 0.534	0.933 0.865	0.005* 0.038*	0.818 0.692	0.758 0.710	33.16 32.68

*indicates statistical significance (*p* < 0.05)

*S1*, sensitivity; *S2*, specificity; *AUC*, area under the curve; *STD*, standard; *CI*, confidence interval; *ILD*, interstitial lung disease; *MCRD*, modified coarseness of reticular disease; *LD Index*, low density volume index; *Up. Lung LDI*, upper lung low density volume index; *Lo. Lung LDI*, lower lung low density volume index; *PD15*, 15th percentile density

**Fig. 5:**
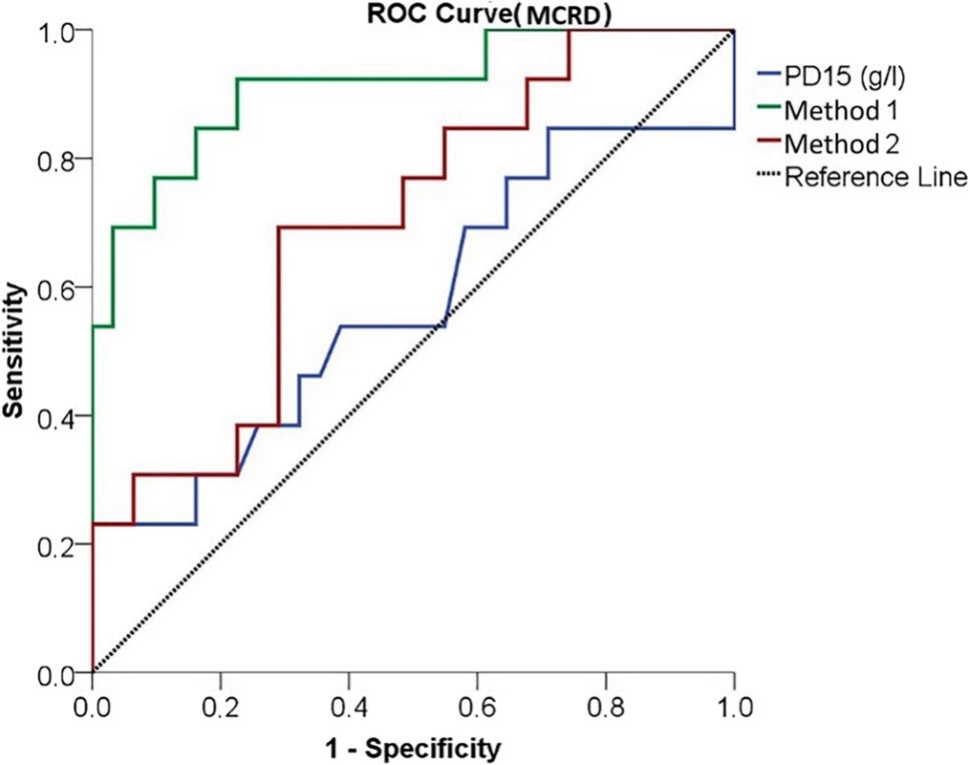
The power of quantitative methods to distinguish limited and extensive disease according to the MCRD score

In terms of the performance of diagnostic tests, the power to distinguish between limited and extensive disease according to the MCRD score, PD15 was found to be weak, while Method 1 and Method 2 were found to be strong. The strongest test was found to be Method 1 (Method 1 AUC = 0.911 *p* < 0.001, Method 2 AUC = 0.700 *p* = 0.038) (Fig. 5, Table 5). When the ROC curves are plotted according to the MCRD score, the cutoff value in distinguishing between limited and extensive disease, it was found to be 14.99 for Method 1 and 32.68 for Method 2 (Table 5).

ROC curve analysis revealed that the quantitative parameters derived from the “Lung Density” program (LDI, Upper Lung LDI, Lower Lung LDI, and Upper/Lower Ratio) demonstrated very limited discriminatory power in distinguishing between limited and extensive disease (Table 5).

Of the 44 patients in the study, 25 had UIP, 16 had NSIP, 2 had LIP, and 1 had DIP pattern. According to ILD involvement patterns, there was no difference between mean values of PFTs and the mean values of QLA methods. There was only one DIP sample, making the calculation impossible.

## Discussion

To our knowledge, this is the first study to show that different QLA methods (Method 1, Method 2, PD15, etc.) can objectively characterize RA-associated ILD compared to two standard semiquantitative methods (ILD and MCRD scores). Our study investigated 7 different quantitative methods. Among seven quantitative methods evaluated, Method 1 showed statistically significant correlations with both semiquantitative assessments and DLCO values in determining RA-ILD severity.

HRCT assessment in RA patients is valuable for ILD diagnosis, classification, monitoring, and treatment response. Correct interpretation of HRCT images and the semiquantitative evaluation of ILD are problems for inexperienced radiologists, rheumatologists, and pulmonologists because there has been significant interobserver variability even among radiologists with experience in thoracic radiology [32, 33]. This is an important problem in the semiquantitative lung assessment (SQLA) of intra- and inter observer variability [34–36]. Although SQLA is calculated from only five CT sections, QLA provides a significant advantage by evaluating the entire lung volume. Through fully automated segmentation, QLA can provide highly accurate and repeatable measurements [37]. The goal of this study was to demonstrate the ability of quantitative methods to enable observer-independent assessment of ILD. Using a single DICOM visualizer (Vitrea) in RA patients, this study is one of the few to show that different QLA methods (Method 1, Method 2) could objectively characterize RA-related ILD more effectively than standard semiquantitative methods like the ILD score and MCRD score.

Quantitative assessment methods present several challenges. There is no standardized cutoff value defined for ILD on the CT histogram in the literature [38]. Marten et al. [13] attempted to determine an ideal threshold for ILD using quantitative CT imaging, resulting in a HU threshold of − 800. Salaffi et al. [10] recommended − 700 HU for lung disease measurement. According to Yabuuchi et al. [9], the most appropriate threshold for measuring ground-glass opacity is − 800 HU. Ninaber et al. [8] proposed percentage density of 85% (Perc85). The cutoff values may also vary for different scanners.

Salaffi et al. [10] calculated the quantitative method, known in our study as Method 1, between − 700 and − 200 HU threshold values in systemic sclerosis patients the OsiriX software. Their results revealed that quantitative results showed a strong correlation with visual-based scoring techniques. Their results revealed significant correlation between PFTs and quantitative method. Our study revealed that semiquantitative visual assessment has a positive correlation with Method 1. Our study revealed that Method 1 has a negative correlation with DLCO and TLC.

Yabuuchi et al. [9] calculated the quantitative method, known as Method 2, between − 800 and − 500 HU to assess ground-glass opacity in SS patients. However, a threshold value of − 800 HU may include small peripheral pulmonary vessels and potentially leading to ILD overestimation [13]. Similarly, in our study, the results showed that Method 2 overestimated ILD.

The − 800 HU threshold effectively segments ground-glass opacities along with reticulation and honeycombing patterns. In contrast, the − 700 HU threshold excludes some ground-glass opacities—potentially including thin reticulation—from segmentation, which may reduce accurary in estimating disease extent [39].

In our study, most patients with RA-associated ILD (*n* = 25, 56.8%) had UIP pattern. No significant differences were observed in QLA, SQLA, or PFT values among patients with NSIP, UIP, or LIP patterns,likely due to small subgroup sample sizes. Therefore, we need to conduct more comprehensive studies to compare the subgroups to the ILD involvement pattern. Furthermore, none of the patients in our study was OP pattern. Peripheral or peribronchovascular consolidations frequently characterize OP, and the study may exclude patients with OP pattern if they do not meet the study criteria.

We recommend PFTs for the evaluation of pulmonary conditions in patients with connective tissue diseases because they are relatively easy to perform. DLCO and FVC have been reported to be mostly correlated by quantitative methods. This may indicate that gas transfer in ILD is more affected than lung compliance [25]. In the literature, DLCO is thought to be most related to the degree of lung involvement. However, DLCO is not a specific biomarker for predicting ILD severity. Other findings such as pulmonary hypertension or anemia may also be the cause of abnormal DLCO measurements. There have also been reports of significant measurement errors in DLCO [11, 24]. The same applies to the FVC. Conflicting results have been reported about the prognostic ability of FVC in ILD [40]. A previous study showed that the degree of lung fibrosis was associated with DLCO but not with FVC [41]. In our study, there was no meaningful correlation between FVC from PFT results and quantitative methods. The significant negative correlation between DLCO and Method 1 or Method 2 appears to be a valuable result.

ILD can affect the quality of life of patients with connective tissue disease. Overall, a few studies reporting quality of life have weak correlations between methods of assessment of quality-of-life [11, 25]. A prior study in a RA cohort reported no statistically significant difference in joint disease activity—as measured by DAS28 scores—between patients with and without ILD [42].We used the DAS28-CRP severity score as a disease severity score in our study. A difference was detected in the means of DAS28-CRP score according to limited and extensive disease obtained by semiquantitative evaluation (*p* = 0.003 for the ILD score and *p* = 0.012 for the MCRD score). Interestingly, disease severity scores were statistically higher for limited diseases than for extensive diseases.

The evaluation of RA-ILD currently relies on subjective visual CT scoring systems that demonstrate interobserver variability, along with PFTs that have significant limitations including late detection of disease progression. Our study developed Quantitative Method 1, which demonstrates strong correlation with DLCO and TLC measurements, providing an early and objective means to detect gas exchange impairment before the decline in FVC becomes apparent. This advancement enables clinical rheumatologists to intervene more proactively in disease progression. Importantly, we determined that the − 700 to − 200 HU range demonstrates higher specificity for early fibrosis compared to − 800 HU thresholds, thereby reducing false positive results. This finding will enable more accurate disease phenotyping. The proposed Method 1 establishes a standardized framework for classifying disease severity (e.g., limited/extensive fibrosis), which can guide both patient selection for clinical trials and decisions regarding antifibrotic therapy initiation. An additional benefit of quantitative methods is their reduced dependence on radiologists through automated analysis tools. This feature is particularly valuable in resource-limited settings, allowing rheumatologists to interpret CT findings more efficiently.

To assess statistical power, post hoc power analyses and effect sizes were computed. The results indicated that the statistical power of the observed differences was generally high. Consequently, despite the limited sample size, the clinical significance of the findings remained substantial. However, in Table 3, Method 2 yielded a power value of 0.669, which falls below the conventionally accepted threshold (typically 0.80) for adequate statistical power in many studies. Although the group sizes were small, the post hoc power analysis demonstrated that most comparisons exhibited sufficient statistical significance, effect sizes, and clinical relevance.

This study has some limitations. First, our study is a retrospective design. The second limitation is that the study population is relatively small, and it is a monocentric study. It will be appropriate to verify our data with multicenter, prospective studies and investigate the applicability of these results.

## Conclusion

Our study shows that quantitative CT assessment methods, especially Method 1, offer a reliable and objective alternative to visual assessment for evaluating the severity of RA-associated ILD. The strong correlation between Method 1 and DLCO, TLC, and visual scoring systems supports its potential clinical benefits in disease monitoring and treatment decision-making.

Method 1 (− 700 to − 200 HU) showed superior performance compared to Method 2 (− 800 to − 500 HU) in terms of better correlation with clinical parameters and less overestimation of disease extent. Quantitative methods eliminate interobserver variability found in semiquantitative assessments. The negative correlation between DLCO and both quantitative methods suggests that these CT-based measurements may reflect the disease’s impact on gas exchange. Threshold selection significantly affects quantitative assessment accuracy. The − 700 HU threshold may provide more specific evaluation than − 800 HU.

The potential for earlier, more consistent ILD detection, the ability to monitor disease progression, and reduced dependence on specialist expertise for interpretation are reasons to prefer quantitative methods.

Based on study limitations, we recommend multicenter prospective validation with larger sample groups. Future research may include investigating correlations with histological findings and examining quantitative methods’ responsiveness to treatment effects.

These findings position quantitative CT analysis as a promising tool for overcoming current challenges in RA-ILD assessment, particularly regarding standardization. The described methods may facilitate more precise phenotyping of ILD severity in clinical practice and research settings.

## Data Availability

All data are kept in the data center of Sütçü İmam University.

## Abbreviations

AUC: Area under the curve
CRP: C-reactive protein
CT: Computed tomography
CTDIvol: Computed tomography dose-index volume
DAS: Disease activity score
DIP: Desquamative interstitial pneumonia
DLCO: Diffusion capacity of the lungs for carbon monoxide
DLP: Dose-length product
FEV: Forced expiratory volume
FEV_1_: Forced expiratory volume in 1 second
HRCT: High-resolution computed tomography
HU: Hounsfield unit
ILD: Interstitial lung disease
LD: Low density
LIP: Lymphocytic interstitial pneumonia
MCRD: Modified coarseness of reticular disease
NSIP: Non-specific interstitial pneumonia
OP: Organizing pneumonia
PD15: 15th percentile density
PFTs: Pulmonary function tests
QLA: Quantitative lung assessment
RA: Rheumatoid arthritis
ROC: Receiver operating characteristics
SQLA: Semiquantitative lung assessment
TLC: Total lung capacity
UIP: Usual interstitial pneumonia

## Declarations

### Originality statement

The revised manuscript contains no plagiarized text passages, duplicated graphics, or previously published material without proper attribution.
